# X‐linked inhibitor of apoptosis inhibition sensitizes acute myeloid leukemia cell response to TRAIL and chemotherapy through potentiated induction of proapoptotic machinery

**DOI:** 10.1002/1878-0261.12146

**Published:** 2017-12-01

**Authors:** Jianbiao Zhou, Xiao Lu, Tuan Zea Tan, Wee‐Joo Chng

**Affiliations:** ^1^ Cancer Science Institute of Singapore National University of Singapore, Centre for Translational Medicine Singapore; ^2^ Department of Medicine Yong Loo Lin School of Medicine National University of Singapore Singapore; ^3^ Translational Centre for Development and Research National University Health System Singapore Singapore; ^4^ Department of Hematology‐Oncology National University Cancer Institute, NUHS Singapore Singapore

**Keywords:** acute myeloid leukemia, apoptosis, BIR2 domain, chemotherapy, TRAIL, XIAP

## Abstract

Acute myeloid leukemia (AML) is an aggressive disease with an increasing incidence and relatively low 5‐year survival rate. Unfortunately, the underlying mechanism of leukemogenesis is poorly known, and there has been little progress in the treatment for AML. Studies have shown that X‐linked inhibitor of apoptosis (XIAP), one of the inhibitors of apoptosis proteins (IAPs), is highly expressed and contributes to chemoresistance in AML. Hence, a novel drug, RO6867520 (RO‐BIR2), developed by Roche targeting the BIR2 domain in XIAP to reactivate blocked apoptosis, is a promising therapy for AML. The monotherapy of RO‐BIR2 had minimal effect on most of the AML cell lines tested except U‐937. In contrast to AML cell lines, in general, RO‐BIR2 alone has been shown to inhibit the proliferation of primary AML patient samples effectively and induced apoptosis in a dose‐dependent manner. A combination of RO‐BIR2 with TNF‐related apoptosis‐inducing ligand (TRAIL) led to highly synergistic effect on AML cell lines and AML patient samples. This combination therapy is capable of inducing apoptosis, thereby leading to an increase in specific apoptotic cell population, along with the activation of caspase 3/7. A number of apoptotic‐related proteins such as XIAP, cleavage of caspase 3, cleavage of caspase 7, and cleaved PARP were changed upon combination therapy. Combination of RO‐BIR2 with Ara‐C had similar effect as the TRAIL combination. Ara‐C combination also led to synergistic effect on AML cell lines and AML patient samples with low combination indexes (CIs). We conclude that the combination of RO‐BIR2 with either TRAIL or Ara‐C represents a potent therapeutic strategy for AML and is warranted for further clinical trials to validate the synergistic benefits in patients with AML, especially for the elderly who are abstaining from intensive chemotherapy.

**Abbreviations**
RO‐BIR2: BIR2 inhibitor RO6867520
TRAIL: TNF‐related apoptosis‐inducing ligand
XIAP: X‐linked inhibitor of apoptosis

## 1 Introduction

Acute myeloid leukemia (AML) is a heterogeneous neoplasia which can be classified into different subtypes based on the clinical features or underlying molecular characteristics (De Kouchkovsky and Abdul‐Hay, 2016; Zhou and Chng, 2017). Despite decade‐long efforts on basic and clinical research, the current mainstay of treatment is still chemotherapy but the outcome of AML remains poor (Tamamyan *et al*., 2017; Zhou *et al*., 2015). The development of targeted therapies holds promise for more effective treatments with reduced side effects (Hackl *et al*., 2017; Zhou *et al*., 2009).

Apoptosis is a mode of cell death used by multicellular organisms to dispose of unwanted cells in various settings. Inappropriate regulation of apoptosis may cause diseases, while evasion of apoptosis is an important hallmark of cancer (Del Poeta *et al*., 2003, 2008; Fathi *et al*., 2010; Li *et al*., 2015b). There are two main pathways leading to the initiation of apoptosis: the mitochondrial‐mediated pathway (intrinsic pathway) and the death receptor‐mediated pathway (extrinsic pathway) (Li *et al*., 2015a; Wallach *et al*., 2008). The convergence of these two pathways is the activation of a family of cysteine proteases (caspases), which ultimately leads to cell death (Aziz *et al*., 2014). The activated caspase 9 (initiator caspase) will mark the initiation of the downstream caspase cascade, causing further proteolytic activation of effector caspases (caspases 3 and 7), which will in turn act on multiple substrates and ends in the eventual apoptosis. To counteract the effect of caspases, mammalian cells are armed with a class of 8 proteins that could inhibit the activity of these enzymes, known as the inhibitor of apoptosis proteins (IAPs) (Clem *et al*., 1996; Hay *et al*., 1995). Among them, the XIAP (X‐linked inhibitor of apoptosis) protein is not only the most potent inhibitor of caspases, but also the only IAP with a direct inhibitory activity on both initiator and effector caspases (Huang *et al*., 2001; Riedl *et al*., 2001).

X‐linked inhibitor of apoptosis (also called BIRC4/IAP‐3) contains 3 tandem repeats called baculovirus inhibitor of apoptosis protein repeat (BIR) at its N‐terminal end, which are evolutionarily conserved across different species (Sun *et al*., 1999). The linker region after BIR domain, together with BIR2 domain, inhibits the activity of effector caspase 3 and caspase 7 through exclusive binding affinity (Huang *et al*., 2001; Riedl *et al*., 2001; Sun *et al*., 1999; Suzuki *et al*., 2001), while BIR3 domain inhibits the activity of initiator caspase 9 by preventing its homodimerization (Shiozaki *et al*., 2003). The inhibitory activity on a family of caspase protein exerted by both linker‐BIR2 and BIR3 domains is suspected to be the cause of the block in apoptosis of cancer cells. Thus, designing a drug that inhibits XIAP activity would provide an enticing prospect in priming cancer cells to their own cell death programs (Baig *et al*., 2016; Lemke *et al*., 2014). In AML, it has been well documented that high expression of XIAP associates with poor overall survival and drug resistance to chemotherapy, or targeted therapy in adults and children.

Clearly, XIAP participates in many processes of AML pathogenesis, and hence, exploiting XIAP as a therapeutic target has attracted great interests among pharmaceutical companies. While the majority of small‐molecule XIAP inhibitors are developed to target BIR3 domain, progress on targeting BIR2 domain is limited. Herein, we investigated the BIR2 inhibitor, RO‐BIR2, which was developed by Roche. This study reports the effects of RO‐BIR2 as single agent or in combination with either TNF‐related apoptosis‐inducing ligand (TRAIL) or chemotherapy on a panel of AML cell lines, primary human AML cells.

## 2 Materials and methods

### 2.1 Cell culture

Human AML cell lines OCI‐AML2 (NPM‐wt), OCI‐AML3 (NPM‐mutant), and MV4‐11 were obtained from DSMZ (Braunschweig, Germany). HL‐60, KG‐1, and U‐937 cell lines were purchased from ATCC (Manassas, VA, USA). OCI‐AML2 and OCI‐AML3 cells were cultured with 80% MEM‐α (GIBCO™, Waltham, MA, USA) with the addition of 20% FBS (JRH Bioscience Inc., Kansas, MO, USA) at density of 0.5–1.5 × 10^6^ cells·mL^−1^. HL‐60, MV4‐11, KG‐1, THP‐1, and U‐937 cells were cultured with RPMI 1640 (Biowest, Nuaillé, France) supplemented with the addition of 10% FBS at the density of 2–10 × 10^5^ cells·mL^−1^ in a humid incubator with 5% CO_2_ at 37 °C.

AML patient samples were obtained from National University Hospital in Singapore and cultured with RPMI 1640 supplemented with 10% FBS and 10 ng·mL^−1^ cytokines (FLT‐3 ligand, IL‐3, CSF, GM‐CSF, TPO from PeproTech, Rocky Hill, NJ, USA) at the density of 5 × 10^5^ cells·mL^−1^ in a humid incubator with 5% CO_2_ at 37 °C. Clinical samples were obtained after receiving informed consent from the patients. The collection of patient samples for research was approved by the Institution's Review Board.

### 2.2 Drug treatment, XIAP ASO, and cell viability assays

BIR2‐specific XIAP inhibitor RO‐BIR2 (RO6867520) was generously provided by Hoffmann‐La Roche AG (Indianapolis, IN, USA). RO‐BIR2 was reconstituted in DMSO to a stock concentration of 10 mm and stored at −20°. Leukemic cells were seeded in 96‐well culture plates at a density of 2 × 10^4^ viable cells/100 μL/well in triplicate. Phosphorothioate XIAP antisense oligonucleotide (ASO) and scrambled ASO were synthesized and purified Integrated DNA Technologies (IDT Singapore). The base compositions of the XIAP antisense and scrambled oligomers were reported (Amantana *et al*., 2004). XIAP ASO and scrambled ASO at various concentration were delivered into the U‐937 cells through electroporation method by the Neon Transfection System (Thermo Fisher Scientific, Waltham, MA, USA) with transfection parameter: 1300 V, 2 pulse, 10 ms. CellTiter‐Glo^®^ Luminescent Cell Viability Assay (CTG assay, Promega, Madison, WI, USA) was used to determine the cell growth and viability as previously described (Zhou *et al*., 2008b). Each experiment was performed in triplicate. IC_50_ was determined by CellTiter‐Glo assay and calculated with compusyn software (Combo Syn, Inc., Paramus, NJ, USA).

### 2.3 Combination index calculation

The compusyn software was used to analyze the dose–effect relationships in AML cell lines and patient samples. The combination index (CI) was applied to quantitatively describe synergism as a greater‐than‐additive effect from a combination of two agents (CI < 1); antagonism as a less‐than‐additive effect (CI > 1); and as an additive effect (CI = 1). The CI values were calculated according to the levels of growth inhibition (fraction affected, Fa) by each agent individually and a combination of different drug compounds (Zhou *et al*., 2008b).

### 2.4 Caspase activity assay and TUNEL assay

To assess the activity of caspase family proteins 24 hr post‐treatment in 96‐well plate, Caspase‐Glo^®^ 3/7 assay and Caspase‐Glo^®^ 8 assay (Promega) were used according to the manufacturer's instruction. TUNEL test (4810‐30‐K, R&D Systems, Minneapolis, MN) was conducted on fixed U‐937 cells treated with DMSO or RO‐BIR2 according to the manufacturer's protocol. Apoptosis was assessed by the TUNEL assay and quantified as a percentage of total cells as previously described (Zhou *et al*., 2008a).

### 2.5 Protein extraction and western blotting

Cells were harvested and lysed with radioimmunoprecipitation assay (RIPA) buffer (20 mm HEPES at pH 7.4, 1% Triton X‐100, 150 mm NaCl, 1 mm EDTA, 1 mm EGTA, and 1 × protease arrest). Total protein concentrations were determined by the Bio‐Rad protein assay (Bio‐Rad Laboratories Inc., Hercules, CA, USA). 10–12% SDS gel was used. Caspase 3, cleaved caspase 3, caspase 7, cleaved caspase 7, and cleaved PARP antibodies were purchased from Cell Signaling Technology (Beverly, MA, USA). XIAP antibody was purchased from R&D systems. Beta‐actin HRP‐conjugated antibody was purchased from Santa Cruz Biotechnology (Santa Cruz, CA, USA). The images were captured using Konica Medical SRX‐101A Medical Film Processor (Konica Minolta Medical & Graphic, Inc., Wayne, NJ, USA). Densitometric analysis was performed using Amersham Image Scanner with labscan imagequant tl Software (Amersham Biosciences, Piscataway, NJ, USA) (Zhou *et al*., 2017). The protein levels of each molecule were normalized with each respective actin level.

### 2.6 Flow cytometric analysis

For apoptosis assays, cells were cultured in 6‐well plates in the presence of different concentrations of RO‐BIR2, TRAIL, or cytarabine (Ara‐C). After 48‐hr incubation, cells were stained with FITC Annexin V Apoptosis Detection Kit 1 (BD Pharmingen™, Thermo Scientific) and SYTOX Blue Dead Cell Stain (Thermo Scientific). The stained cells were immediately analyzed using LSRII flow cytometer (BD Biosciences, San Jose, CA, USA) and flowjo software (TreeStar Inc, Ashland, OR, USA).

### 2.7 Statistical analysis

The experimental results presented in the figures in the Results section were representative of duplicate or triplicate observations. The data were presented as the mean values ± standard derivation of the mean (SD). Comparisons between two groups were evaluated by *t*‐test. Multiple comparisons of more than two groups were evaluated by two‐way analysis of variance (ANOVA). For FAB, *P*‐value is computed by ANOVA test, followed by Tukey's multiple comparison test. For age correlation, Spearman's correlation was used to compute *P*‐value. Values of *P *≤* *0.05 were considered to be statistically significant. All statistical tests were performed using graphpad prism, version 4 (La Jolla, CA, USA).

## 3 Results

### 3.1 RO‐BIR2 inhibits proliferation of primary AML cells and selective AML cell lines

**Figure 1:**
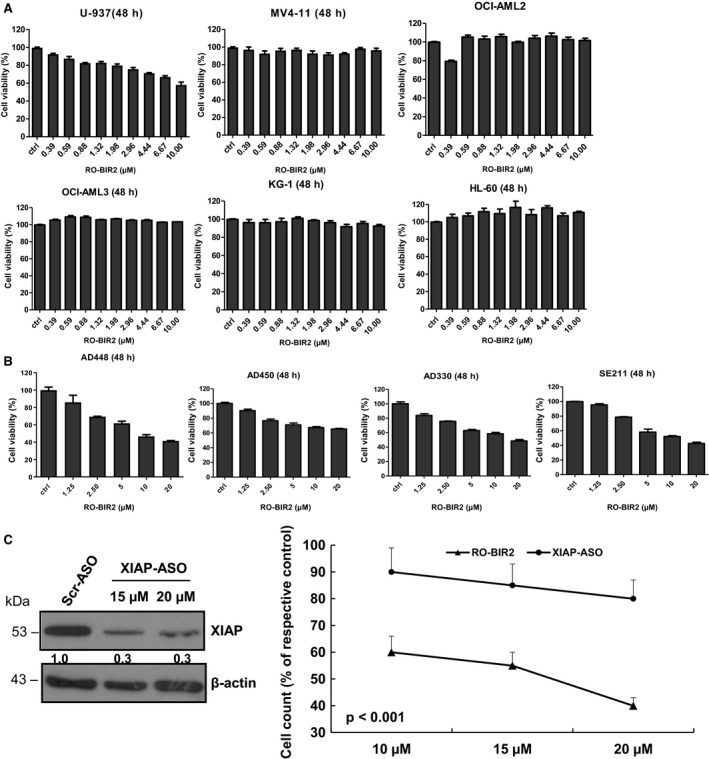
Cell proliferation assay analysis of RO‐BIR2 monotherapy on AML cell lines (A) and primary AML cells (B). AML cell lines including U‐937, MV4‐11, OCIA‐ML2, OCI‐AML3, KG‐1, HL‐60 and primary bone marrow cells from patients AD448, AD450, AD330, and SE211 were incubated with either DMSO control or increasing concentration of RO‐BIR2 for 48 h, followed by CTG assays. The treated results were shown in percentage after normalization with their DMSO control (100%), respectively (*n* = 3, mean ± SD). (C) Effects of XIAP ASO and RO‐BIR2 on U‐937 cells. Left panel: immunoblotting analysis of lysates from cells treated with XIAP ASO and scrambled ASO after transfection for 48 h. Beta‐actin was used as loading control. Protein levels were determined by densitometric analysis. Right panel: comparison of XIAP ASO and RO‐BIR2 on cell proliferation of U‐937 cells. Same number of U‐937 cells treated with either XIAP ASO or scrambled (Scr) ASO (control), RO‐BIR2 or DMSO (control) for 48 h, followed by CTG assays. The results were expressed as means of triplicate values (± SD) relative to Scr‐ASO or DMSO control (*P* < 0.001).

We first investigated the effects of RO‐BIR2 on the growth of six human AML cell lines, representing different FAB subtypes. RO‐BIR2 drug was treated on six AML cells at concentrations of 10 μm to 0.39 μm (1.5 times dilution) for 48 h and DMSO was used as control. The results from the CTG assay showed a gradual decrease in cell proliferation along with progressive increases in the concentrations of RO‐BIR2 over 48 h in U‐937 cell lines, while the other AML cell lines (KG‐1, OCI‐AML2, OCI‐AML3, MV4‐11, HL‐60) were relatively resistant to RO‐BIR2 (Fig. 1A). In contrast, all primary AML blasts from four patients (AD450, AD330, AD448, and SE211) were sensitive to RO‐BIR2 monotherapy, showing dose‐dependent inhibition on cell growth (Fig. 1B). Overall, these data indicate the different responses to inhibition of XIAP‐BIR2 between AML primary cells and cell lines.

Next, we compared the activities of RO‐BIR2 and XIAP ASO. Phosphorothioate XIAP ASO and scrambled ASO at various concentrations were delivered into the U‐937 cells through electroporation method. Figure 1C shows that XIAP ASO downregulated endogenous XIAP protein, leading to a moderate decrease (20%) in cell viability compared with scrambled ASO control. The effect of XIAP ASO on cell viability was less potent than that of RO‐BIR2 on U‐937 cells (Fig. 1C, *P* < 0.001).

### 3.2 RO‐BIR2 single agent induces apoptosis of primary AML cells and U‐937 cells

**Figure 2:**
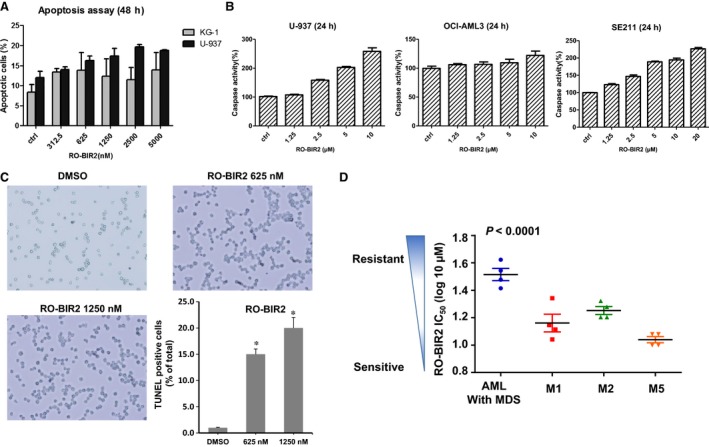
The effect of RO‐BIR2 on induction of apoptosis reactions on AML cell lines and primary AML cells. (A) U‐937 and KG‐1 cells were treated with either DMSO control or RO‐BIR2 at indicated doses for 48 h. Cells were harvested, washed, and stained with Annexin V/SYTOX Blue double dye, then subjected to flow cytometry analysis. The percentage of Annexin V‐positive cells of each cell line was normalized with respective DMSO control. (B) U‐937, OCI‐AML3, and primary bone marrow cells from patient SE211 were treated with either DMSO control or various concentrations of RO‐BIR2 for 24 h, then harvested for caspase 3/7 activity assays. The caspase 3/7 activity was presented to increasing percentage relative to that of DMSO control (100%). All experiments were duplicated, and results were shown as mean ± SD. (C) Detection of apoptosis by TUNEL assay in U‐937 cells in response to RO‐BIR2. Duplicated experiments were conducted and representative images were shown. The bar figure represented the quantification of apoptotic cells over total number of cells. Data were mean ± SD (*n* = 3) (*P < 0.01). (D) IC_50_ of 16 primary AML samples tested in 48‐h cell proliferation assays (CTG) and grouped according to FAB subtype (French–American–British classification of AML cells). Results show mean ± SD from triplicates of experiments. AML with MDS: AML with MDS history or phenotypic changes (*P* < 0.0001 versus M1, M2, or M5).

We next determined the apoptotic effect of RO‐BIR2 on AML cell lines and primary AML cells. KG‐1 and U‐937 cells were incubated with increasing concentration of RO‐BIR2 for 48 h, followed by FACS analysis of apoptotic populations. While single treatment of RO‐BIR2 did not lead to any significant changes in apoptotic KG‐1 cell population, U‐937 cells showed a dose‐dependent response to RO‐BIR2 with increasing cell death (Figs 2A and S1). Caspase 3 and caspase 7 are executioner caspases for the apoptosis intrinsic pathway that drive the apoptosis pathway directly, and they can be inhibited by the XIAP linker‐BIR2 domain (Kaufmann *et al*., 2012). Consistent with the CTG results above, a dose‐dependent increase in caspase 3/7 activity was observed in U‐937 cell and SE211 primary cells, but not in OCI‐AML3, which is resistant to RO‐BIR2 after treatment for 24 h (Fig. 2B). Furthermore, TUNEL assay revealed a significant induction of cellular apoptosis in the RO‐BIR2‐treated U‐937 cells (Fig. 2C). Taken together, these results suggest that RO‐BIR2 monotherapy promotes apoptosis in sensitive AML cells through activation of caspases 3 and 7. To further investigate the correlation of clinicopathological characteristics with RO‐BIR2 sensitivity, we treated these cells with RO‐BIR2 and then used CTG assay to determine their viability and calculated their IC_50_ in 16 primary AML samples. RO‐BIR2 IC_50_ ranged from 10 to 42 μm (Table 1). We detected that patients diagnosed with AML with MDS (myelodysplastic syndromes) were significantly resistant to RO‐BIR2 (median 32.5 μm,* P* < 0.0001 when compared to all other groups). This result is consistent with clinical observation that AML with MDS changes is a subentity that has a poor prognosis (Vardiman and Reichard, 2015). Interestingly, similar to the cell lines, a group of FAB‐M5 AML patients were more sensitive to RO‐BIR2 (median 11 μm), followed by samples with FAB‐M1 (median 13.5 μm) and FAB‐M2 (median 16 μm) (Fig. 2D). In addition, we found that the RO‐BIR2 sensitivity did not correlate with FLT3 mutation (*P* = 0.14), NPM mutation (*P* = 0.46), karyotype (*P* = 0.34), sex (*P* = 0.32), or age (*P* = 0.64).

**Table 1:** Clinical characteristic of 16 AML patients and their IC_50_ for RO‐BIR2

Patient ID	Sex	Age (years)	FAB	Karyotype	FLT3	NPM1	IC_50_ (RO‐BIR2), μm
AD330	M	56	M2	Normal	FLT3‐ITD	Mutant	16
AD448	M	74	M5	Normal	N.A.	N.A.	10
AD450	M	62	AML with MDS	Normal	Wild‐type	Mutant	42
SE211	M	79	M1	47, XY, +11	Wild‐type	Wild‐type	11
Patient 5	F	41	M1	Normal	Wild‐type	Wild‐type	13
Patient 6	F	49	M1	Normal	FLT3‐ITD	Mutant	22
Patient 7	F	65	M2	t(8;21)	FLT3‐ITD	N.A.	19
Patient 8	M	42	AML with MDS	47,XY,+8	Wild‐type	Wild‐type	30
Patient 9	F	53	M5	Complex Karyotype	FLT3‐ITD	N.A.	12
Patient 10	F	66	M1	47,XX,+11	Wild‐type	N.A.	14
Patient 11	F	52	M5	Normal	FLT3‐ITD	Mutant	10
Patient 12	F	62	AML with MDS	Normal	Wild‐type	Wild‐type	26
Patient 13	M	54	M2	47,XX,+8	FLT3‐ITD	Mutant	16
Patient 14	M	62	AML with MDS	Normal	Wild‐type	Mutant	35
Patient 15	F	42	M2	Normal	FLT3‐ITD	Mutant	21
Patient 16	F	45	M5	47,XX,+8	FLT3‐ITD	Wild‐type	12

M, male; F, female; y, years; N.A., not available.

### 3.3 Combination therapy of RO‐BIR2 with TRAIL produces synergetic antileukemic effect on AML cells

**Figure 3:**
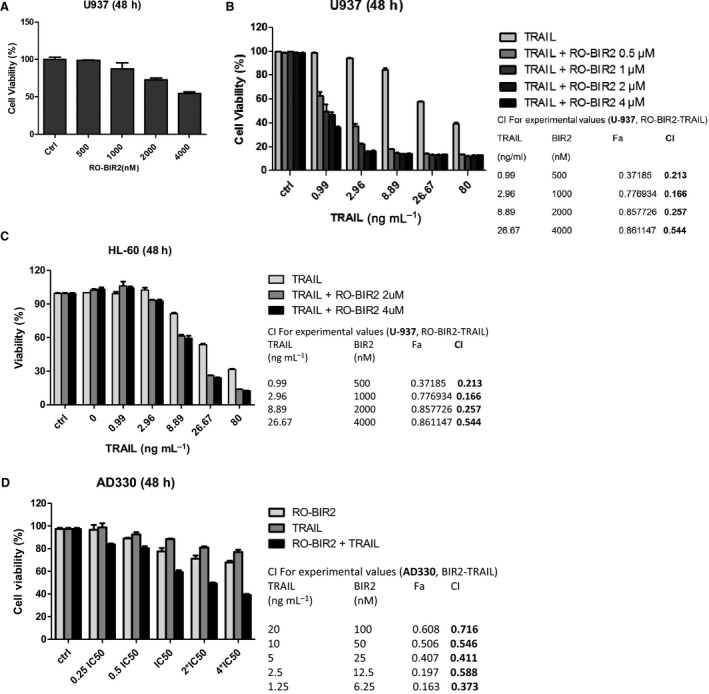
Combination of RO‐BIR2 and TRAIL synergistically inhibits proliferation of AML cells. (A) U‐937 cells were treated with TRAIL single agent for 48 h. (B) U‐937 cells, or (C) HL‐60 cells, or (D) AD330 primary cells were treated for 48 h with either single agent (RO‐BIR2 or TRAIL alone) or in combination at the indicated concentrations. Cell viability was then analyzed by CTG assays (*n* = 3, mean ± SD). CI values as calculated from experiments were shown side by side of each bar graph. CI<1 represents synergistic effect.

TNF‐related apoptosis‐inducing ligand (TRAIL), a member of the TNF superfamily, has been shown to induce apoptosis in many cancer cells through the activation of extrinsic apoptosis pathway (de Miguel *et al*., 2016; Tazzari *et al*., 2008). However, a large number of TRAIL‐based clinical trials conducted so far have limited success owing to the cancer cells having primary or developing secondary resistance to TRAIL‐induced apoptosis (Dimberg *et al*., 2017). Thus, a potent sensitizer of TRAIL‐related therapy is much needed in the clinic. We first studied whether canonical TRAIL signaling is intact in the AML cells; we set to quantitate three TRAIL‐induced genes, that is, IL‐8, E‐selectin, and BNIP3, in U937 and HL60 cells upon exposure with TRAIL (Liu *et al*., 2013; Wang *et al*., 2015). As shown in Fig. S2, qRT‐PCR analysis demonstrated upregulation of these three genes by TRAIL treatment. These results suggest that TRAIL‐mediated response in AML cells functions well. Next, to examine whether RO‐BIR2 acting on intrinsic pathway has a synergistic effect with TRAIL, one sensitive cell line (U‐937) and one resistant cell line (HL‐60) were used. When given as a single agent, TRAIL treatment led to a dose‐dependent inhibition of proliferation in U‐937 cells (Fig. 3A). When TRAIL was combined with low concentrations of RO‐BIR2 at 0.5 μm, 1 μm, 2 μm, and 4 μm, the cell viability decreased significantly compared to TRAIL single treatment as confirmed by two‐way ANOVA (Fig. 3B). The combination effect of TRAIL and RO‐BIR2 was analyzed by CompuSyn to calculate the CI values. These combinations produced strong synergisms at the first three combinations (CI < 0.3) and synergism (CI < 0.6) at the fourth combination (the highest dose combination) in U‐937 cells (Fig. 3B).

We next assessed the combination effect of TRAIL and RO‐BIR2 on HL‐60, a RO‐BIR2‐resistant line. As shown in Fig. 3C, treatment of RO‐BIR2 at 2 μm and 4 μm had little effect on cell viability (the plot above TRAIL 0 ng**·**mL^**−1**^) as previously demonstrated, while incubation with TRAIL impeded HL‐60 cell survival in a dose‐dependent fashion. Notably, the combined treatment was much more cytotoxic than either of the single treatment. All the combinations gave an effect which ranged from synergistic (CI < 0.6) to strong synergistic (CI = 0.3; Fig. 3C). Two‐way ANOVA was performed to confirm the significant difference between combination treatment and single treatment (Fig. 3C).

The synergistic effect of RO‐BIR2 and TRAIL on inhibition of cell survival was also examined in one primary AML sample AD330, which exhibited dose‐dependent response to both drugs. With that, we calculated IC_50_ of these two drugs and used the constant ratio combination of both: 0.25 × IC_50_, 0.5 × IC_50_, 1 × IC_50_, 2 × IC_50_, and 4 × IC_50_. Samples subjected to combined treatment recorded a significantly lower cell viability percentage as compared to samples treated with single‐agent RO‐BIR2 or TRAIL. There was a significant difference between the two sets of treatment (*P* < 0.001, analyzed by two‐way ANOVA) (Fig. 3D). The calculated CI values ranging from 0.4 to 0.7 also confirmed the synergism of this combination therapy on patient sample AD330 (Fig. 3D).

Taken together, these results indicate that RO‐BIR2 and TRAIL can act synergistically to decrease the proliferation of AML cells regardless of their pre‐existing susceptibility to RO‐BIR2.

### 3.4 Enhanced cell death of AML cells by combination of RO‐BIR2 and TRAIL

**Figure 4:**
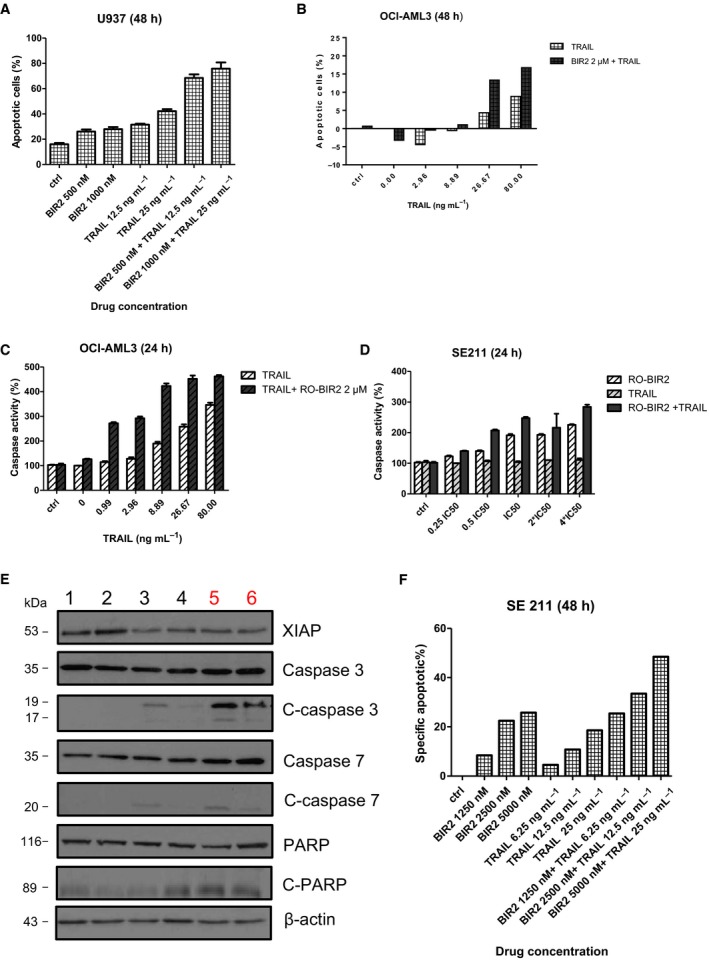
Combination of RO‐BIR2 and TRAIL enhances cell death of AML cells. FACS analysis of apoptotic population in U‐937 (A) or OCI‐AML3 (B) cells treated with either RO‐BIR, TRAIL single agents, or combination (*n* = 2, mean ± SD). Luminescent assays for caspase 3 and caspase 7 activities in OCI‐AML3 cells (C) or primary AML cells from sample SE211 (D) incubated with either RO‐BIR, TRAIL single agents, or combination at 24 h (*n* = 3, mean ± SD). (E) Western blot analysis for XIAP, caspase 3, cleaved (C) caspase 3, caspase 7, C‐caspase 7, PARP, and C‐PARP in OCI‐AML3 cells with different regimes as indicated. Lane 1, DMSO control; lane 2, RO‐BIR2 2 μm; lane 3, TRAIL 20 ng·mL^−1^; lane 4, TRAIL 10 ng·mL^−1^; lane 5, RO‐BIR2 2 μm+TRAIL 20 ng·mL^−1^; lane 6, RO‐BIR2 2 μm+ TRAIL 10 ng·mL^−1^. Beta‐actin served as loading control. Protein levels were determined by densitometric analysis. The experiments were duplicated and representative images were shown. (F) FACS analysis of apoptotic population in primary AML cells from SE112 case treated with either RO‐BIR, TRAIL single agents, or combinations (*n* = 2, mean ± SD).

Given that RO‐BIR2 and TRAIL activate the intrinsic and extrinsic apoptosis pathways, respectively, we evaluated whether this combined modality could stimulate the synergism of induction of cell death of U‐937 (sensitive) and OCI‐AML3 (resistant) AML cells. In U‐937 cells, neither of the single agent induced apoptosis, but combination therapy resulted in a much higher percentage of apoptotic cells (Fig. 4A). With a constant ratio combination of a low dose of RO‐BIR2 (500 nm) and TRAIL (12.5 ng·mL^−1^), the apoptotic cell population reached 50% of U‐937 cells (Fig. 4A). In OCI‐AML cells, the FACS analysis revealed that only TRAIL single treatment could increase specific apoptotic cell population, while RO‐BIR2 alone has limited effect. Importantly, with the addition of RO‐BIR2, the apoptotic response to TRAIL was significantly enhanced as a higher number of OCI‐AML3 cells were found to undergo apoptosis (Figs 4B and S3).

The combination therapy is associated with an increased activity of caspase 3/7 in OCI‐AML3 and U‐937 cells. A strong activation of caspase 3/7 around 300% was observed in OCI‐AML3 cells when RO‐BIR2 was used in combination with low doses of TRAIL (0.99 ng·mL^−1^ and 2.96 ng·mL^−1^), while the TRAIL single‐treated sample only showed caspase 3/7 activity of 100‐130% (Fig. 4C). We also performed caspase 3/7 assay in primary AML cells exposed to the combination therapy. TRAIL monotherapy has very limited effect on caspase 3/7 activity in SE211 cells as the caspase activity remained around 100% in the presence of all doses of TRAIL tested from low to high concentrations (Fig. 4D). On the other hand, caspase 3/7 activity increases with higher RO‐BIR2 concentration. However, when RO‐BIR2 was combined with TRAIL, the caspase 3/7 activity became much higher and the significance of this increase was confirmed by two‐way ANOVA (Fig. 4D).

Consistent with the results from FACS analysis and caspase 3/7 assays, western blot analysis showed that RO‐BIR2 monotherapy could not lead to perceptible change in XIAP, caspase 3, cleaved caspase 3, caspase 7, cleaved caspase 7, PARP, and cleaved PARP. Notably, western blot analysis indicated that XIAP protein level was reduced, while cleavages of caspases 3, 7 and PARP were increased in OCI‐AML3 cells after incubation with RO‐BIR2 and TRAIL for 48 h (Fig. 4E). We further validated the effect of combination of RO‐BIR2 and TRAIL on AML patient samples by constant ratio combination therapy in SE211 cells. As shown in Fig. 4F, RO‐BIR2 single treatment at the concentration of 5 μm could induce more than 20% apoptotic cell population, but TRAIL alone was not effective. Impressively, combination of lower concentration of RO‐BIR2 at 1.25 μm and 6.25 ng of TRAIL incited more than double of the cell death than with RO‐BIR2 TRAIL single treatment (Figs 4F and S3). Overall, the combination treatment has increased apoptotic cell population significantly, enhanced activation of caspase 3/7, magnified cleaved PARP, as well as reduced XIAP protein as compared to RO‐BIR2 or TRAIL single treatment.

In conclusion, RO‐BIR2 maximally potentiates the proapoptotic effect of TRAIL. RO‐BIR2 and TRAIL combination has led to synergistic cell death of AML cell lines and primary AML cells. This synergism in killing is independent of the intrinsic sensitivity to RO‐BIR2. Mechanistically, this combination therapy exerts synergism through the inhibition of XIAP protein, inducing the activity of caspase 3 and caspase 7 and downstream PARP cleavage, leading to final cell death. These results suggest that RO‐BIR2 in combination with low dose of TRAIL is found to be a better therapy and is beneficial for patients with AML.

### 3.5 Combination of RO‐BIR2 with chemotherapy leads to synergistic activity

**Figure 5:**
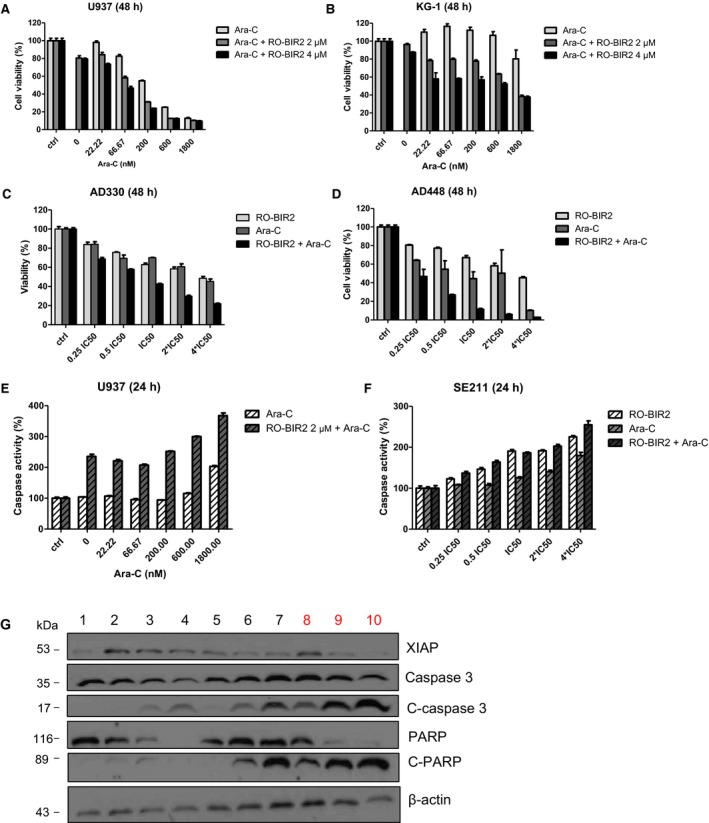
RO‐BIR2 and Ara‐C is synergistic in AML cell lines and primary AML cells. AML cell lines U‐937 (A), KG‐1 (B), and primary bone marrow cells from patients AD330 (C) and AD448 (D) were treated with either RO‐BIR2, Ara‐C alone or in different doses of combination for 48 h, followed by CTG assays. The treated results were shown in percentage after normalization with their DMSO control (100%), respectively (*n* = 3, mean ± SD). Luminescent assays for caspase 3 and caspase 7 activities in U‐937 cells (E) or primary AML cells from sample SE211 (F) incubated with either RO‐BIR, Ara‐C single agents, or combinations for 24 h (*n* = 3, mean ± SD). (G) Western blot analysis for XIAP, caspase 3, cleaved (C) caspase 3, PARP, and C‐PARP in primary AML cells from patient SE112 with various treatments as indicated. Lane 1, DMSO control; lane 2, RO‐BIR2 1.25 μm; lane 3, RO‐BIR2 2.5 μm; lane 4, RO‐BIR2 5 μm; lane 5, Ara‐C 175 nm; lane 6, Ara‐C 350 nm; lane 7 Ara‐C 700 nm; lane 8, RO‐BIR2 1.25 μm + Ara‐C 175 nm; lane 9, RO‐BIR2 2.5 μm + Ara‐C 350 nm; lane 10, RO‐BIR2 5 μm + Ara‐C 700 nm. Beta‐actin was used as loading control. Protein levels were determined by densitometric analysis. The experiments were duplicated and representative images were shown.

Cytarabine (Ara‐C) is one of the most commonly used chemotherapeutic drugs to treat AML and non‐Hodgkin lymphoma (Xie *et al*., 2010). We aimed to interrogate the possible synergism of RO‐BIR2 in combination with Ara‐C, the current backbone drug for patients with AML. With the combination of chemotherapeutic drug Ara‐C, RO‐BIR2 has proven promising inhibitory effect of cell proliferation on both U‐937 cells (RO‐BIR2 sensitive) and KG‐1 cells (RO‐BIR2 resistant) (Fig. 5A, B). As shown in Fig. 5, Ara‐C exerted a dose‐dependent inhibition on U‐937 and KG‐1 cells, and when combined with 2 μm or 4 μm RO‐BIR2, the cell viability decreased significantly compared with either of the single drug treatment (*P* < 0.05). Interestingly, combination therapy of higher dose of 4 μm RO‐BIR2 performed better than that of lower dose of 2 μm RO‐BIR2 (*P* < 0.05). Two‐way ANOVA indicated that combination treatment and single treatment were significantly different in both U‐937 and KG‐1 cells (Fig. 5A,B). Synergistic action was achieved in U‐937 cells from all the combinations as the calculated CI values were < 1 (Table 2). Combination therapy in KG‐1 cells led to a great decrease in cell viability compared with RO‐BIR2 or Ara‐C monotherapy. With KG‐1 cells being less sensitive to Ara‐C drug than U‐937 cells, at 22.22 nm to 600 nm Ara‐C treatment, there was no inhibitory effect but a slight increase in cell viability; thus, the CI value could not be calculated (CompuSyn software requires inhibition value >0; inhibition value <0 could not be entered into the software). Nevertheless, we could still draw the conclusion that the combination of RO‐BIR2 and Ara‐C works effectively on KG‐1 cells, and that even at low doses of Ara‐C, combination therapy could remarkably decrease cell viability as compared to treatment with single agent (Fig. 5B, *P* < 0.05 or *P* < 0.01).

**Table 2:** Combination indexe values of RO‐BIR2 and Ara‐C combination therapy in U‐937 AML cell line and AD448, AD330 primary AML cells (CI < 1, synergistic; CI > 1, antagonistic; CI = 1, additive)

RO‐BIR2 (μm)	Ara‐C (nm)	Effect	CI
U‐937			
2.0	1800.0	0.9	1.09764
2.0	600.0	0.87	0.45962
2.0	200.0	0.69	0.35757
2.0	66.67	0.42	0.28295
4.0	1800.0	0.9	1.09764
4.0	600.0	0.88	0.42837
4.0	200.0	0.76	0.27257
4.0	66.67	0.53	0.20119

RO‐BIR2 (μm)	Ara‐C (nm)	Effect	CI
AD448			
20	2800	0.97	0.20418
10	1400	0.94	0.21631
5	700	0.88	0.23914
2.5	350	0.76	0.34528
1.25	175	0.53	0.44894

RO‐BIR2 (μm)	Ara‐C (nm)	Effect	CI
AD330			
20	2800	0.779	0.29769
10	1400	0.702	0.29117
5	700	0.573	0.37161
2.5	350	0.421	0.51557
1.25	175	0.313	0.56162

Primary bone marrow cells from two patients with AML, AD330 and AD448, were probed for the impact of combination therapy by constant ratio combination of these two drugs. In both AD330 and AD448 cells, RO‐BIR2 in addition to Ara‐C generated greater inhibition on cell proliferation (Fig. 5C,D). We observed remarkable cytotoxicity in AD448 at 48 h after treatment with both drugs. This combination at the lowest dose of 1.25 μm RO‐BIR2 and 175 nm Ara‐C produced inhibitory effect of as high as 0.53 (Fig. 5D). The calculated CI values of less than 0.3 in three combinations indicate a great synergism between these two drugs. The combination also showed good synergism (CI < 1) in AD330 cells (Fig. 5C).

To test whether simultaneous treatment with RO‐BIR2 and Ara‐C has synergism in stimulating apoptosis against AML cells, U‐937 cells were treated with various concentrations of Ara‐C alone or 2 μm RO‐BIR2 or in combination for 24 h. Cells were then subjected to caspase 3/7 activity assays. As shown in Fig. 5E,F, in U‐937 cells, 2 μm of RO‐BIR2 monotherapy had minimum effect on caspase activation. However, when combined with a low dose of Ara‐C (22.22 nm), the caspase activity increased from 100% to more than 200% (Fig. 5E). An even higher caspase activity (>300%) was observed in U‐937 cells co‐incubated with 2 μm of RO‐BIR2 and 1800 nm of Ara‐C. In patient sample SE211, which responded to RO‐BIR2 and Ara‐C dose dependently, combination therapy at constant ratio based on their IC_50_ was performed. Co‐administration of RO‐BIR2 and Ara‐C resulted in synergistic activation of caspases 3 and 7 in primary bone marrow cells from patient SE211 (Fig. 5F).

Efforts were then undertaken to determine the molecular mechanism by which inhibition of XIAP by RO‐BIR2 underlies the synergistic augmentation of cell death between the two agents. Several different concentrations of RO‐BIR2 or Ara‐C single agent and their combination were used to treat SE‐112 cells for 48 h, followed by western blot analysis of XIAP, as well as caspases. Concurrent treatment with RO‐BIR2 significantly augmented apoptosis induction by Ara‐C, as evidenced by escalated cleavage of caspase 3 and cleaved PARP, in association with synergistic downregulation of XIAP protein than control and single‐treated samples (Fig. 5G). These results suggest that potentiated induction of the apoptotic machinery is important for the therapeutic effect of the combination of RO‐BIR2 and Ara‐C. The sensitization of AML cells to Ara‐C by RO‐BIR2 is due to the inhibition of XIAP activity, rather than to off‐target effects. It is known that chemotherapy causes serious side effects in patients with AML. Taken together, we propose that the combination of RO‐BIR2 and Ara‐C might have important clinical benefits as the use of lower dose of Ara‐C could minimize systematic drug toxicity in patients with AML. Well‐planned clinical trials are needed to validate the combination benefits.

## 4 Discussion

It is of great significance for patients with AML to take minimal dosage of any particular drug but still achieve the expected treatment outcome with much less or no toxicity. Optimal combination therapies have proven to reduce not only the side effects, but also the possibility of developing drug resistance. In this study, we investigated the effects of a novel XIAP‐BIR2 inhibitor, developed by Roche AG in AML cells. We focused on the combination of XIAP inhibition with TRAIL, as well as Ara‐C, a main chemotherapy drug currently used to treat AML.

The drug RO‐BIR2 was designed to target XIAP BIR2 domain to activate caspase 3 and caspase 7, thus initiating cell death through the intrinsic pathway. The monotherapy of RO‐BIR2 had minimal effect on most of the AML cell lines tested except U‐937. In contrast to AML cell lines, in general, RO‐BIR2 alone has shown to effectively inhibit the proliferation of primary AML patient samples and induced apoptosis dose dependently.

Combination of RO‐BIR2 with TRAIL or the chemotherapy drug, Ara‐C, has led to highly synergistic effect in AML cell lines and AML patient samples. Combination therapies have induced apoptosis and led to the increase in specific apoptotic cell population, along with the activation of caspase 3/7. A number of apoptotic‐related proteins such as XIAP, cleavage of caspase 3, cleavage of caspase 7, cleaved PARP, etc, were changed upon combination therapy. The data suggest a mechanism by which a potentiated inhibition of XIAP protein is essential for this synergistic effect following combination therapy.

Early phase I/II clinical trials evaluating the effect of a XIAP antisense oligonucleotide (ASO), AEG35156, in combination with chemotherapy demonstrate a better outcome in patients with AML refractory as compared to a single induction regimen (Schimmer *et al*., 2009). However, these optimal results could not be reproduced in the randomized phase II study (Schimmer *et al*., 2011). One common disadvantage of ASO drugs lies in its inability to achieve long‐term suppression of its target, thus limiting their clinical response. Meanwhile, small–molecule inhibitors like RO‐BIR2 can circumvent this drawback, often inactivating their targets rapidly and sustainably. Crystal structure and biochemistry studies revealed that BIR2 domain makes numerous contacts with the caspase 3 and caspase 7. There is particular interest in the development of BIR‐2 selective inhibitor, like RO‐BIR2, in hopes of triggering both of the intrinsic and the extrinsic pathways.

**Figure 6:**
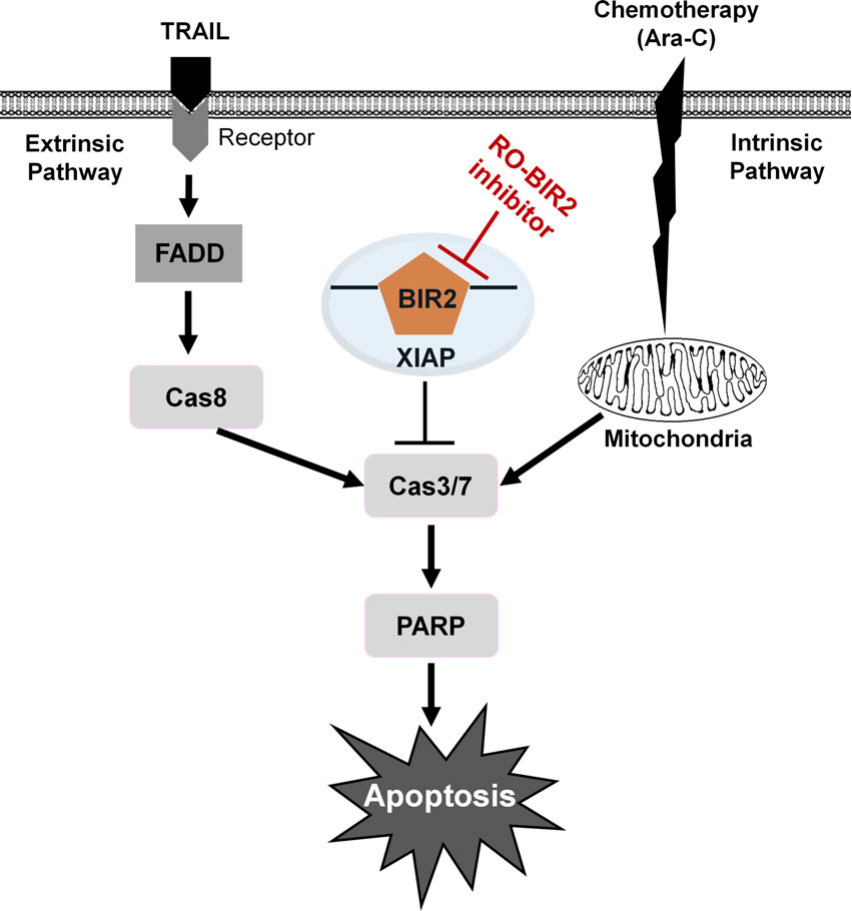
The schematic representation of XIAP inhibitor RO‐BIR2 in combination with TRAIL or Ara‐C in synergistic activation of apoptotic cascade.

Based on the collective results, we proposed a model of RO‐BIR2 combination therapy (Fig. 6). RO‐BIR2 binds to BIR2 domain, and the release of caspase 3 and caspase 7 affects the downstream cleavage of PARP, thus leading to cell death. TRAIL could trigger the extrinsic pathway, and the activation of caspase 8 induces downstream executioner caspase, caspase 3 and caspase 7 and PARP cleavage, and finally leading to apoptosis. Chemodrug Ara‐C functions more on the intrinsic pathway by DNA damage and involves the mitochondria. The cleavage of caspase 3/7 also leads to the downstream PARP cleavage and undergoes apoptosis.

## 5 Conclusions

In summary, the combination of RO‐BIR2 with TRAIL or Ara‐C represents a potent therapeutic strategy for AML, and properly designed clinical trials of these combinations are essential to validate the synergistic benefits in patients with AML, especially for the elderly, who are less tolerable to intensive chemotherapy.

## Author contributions

JZ and W‐JC designed and initiated this study. JZ and XL performed the experiments. TZT performed the bioinformatics analysis. JZ, XL, and W‐JC wrote the manuscript. All authors read and approved the final manuscript.

## Supporting information


**Fig. S1.** FACS analysis of apoptosis on KG‐1 (A) and U‐937 (B) cells treated with RO‐BIR2.
**Fig. S2.** Quantification of TRAIL‐induced genes in U‐937 and HL60 cells by qRT‐PCR.
**Fig. S3.** FACS analysis of specific apoptotic cell population of AML cell line OCI‐AML3 (A) and primary AML cells from patient SE211 (B).
