# Supplementary material for: X‐linked inhibitor of apoptosis inhibition sensitizes acute myeloid leukemia cell response to TRAIL and chemotherapy through potentiated induction of proapoptotic machinery
Source: Mol Oncol. 2017 Dec 1;12(1):33–47. doi: 10.1002/1878-0261.12146 (PMC5748481; doi:10.1002/1878-0261.12146)
Supplement: Supplementary file 1 — Fig. S1. FACS analysis of apoptosis on KG‐1 (A) and U‐937 (B) cells treated with RO‐BIR2. Fig. S2. Quantification of TRAIL‐induced genes in U‐937 and HL60 cells by qRT‐PCR. Fig. S3. FACS analysis of specific apoptotic cell population of AML cell line OCI‐AML3 (A) and primary AML cells from patient SE211 (B). [file MOL2-12-33-s001.docx]

**Supplementary Methods**

**Reverse transcription (RT)-PCR and Real-time quantitative (q) RT-PCR**

One microgram of total RNA from experimental samples was reverse transcribed with Superscript reverse transcriptase (Invitrogen), using oligo(dT) primer for first-strand cDNA synthesis. The sequences of primers of three human TRAIL-induced genes for qRT-PCR were as follows:

IL-8-Forward: 5’-CTCTTGGCAGCCTTCCTGATT-3’

IL-8-Reverse: 5’-ACTCTCAATCACTCTCAGTTCT-3;

E-selectin-Forward: 5’-GAATGTGTAGAGACCATCATAATAAT-3’

E-selectin-Reverse: 5’-AGGAAGAATTGRAGCTGAAGTTT-3’

BNIP3-Forward: 5’-CCGGGATGCAGGAGGAGAG-3’

BNIP3-Reverse: 5’-TTATAAATAGAAACCGAGGCTGGAAC-3’

Sequences of primers of GAPDH for qRT-PCR were published before.^1^ Power SYBR^®^ Green PCR Master Mix was used as recommendation by the manufacturer (Applied Biosystems). GAPDH was used as internal control. SDS 2.2.1 software (Applied Biosystems) was used to perform relative quantitation (RQ) of target genes using the comparative C_T_ (ΔΔC_T_) method.

**Reference**

1. Zhou J, Bi C, Ching YQ, Chooi JY, Lu X, Quah JY*, et al.* Inhibition of LIN28B impairs leukemia cell growth and metabolism in acute myeloid leukemia. *J Hematol Oncol* 2017, **10**(1)**:** 138.

**Supplementary Figure S1**


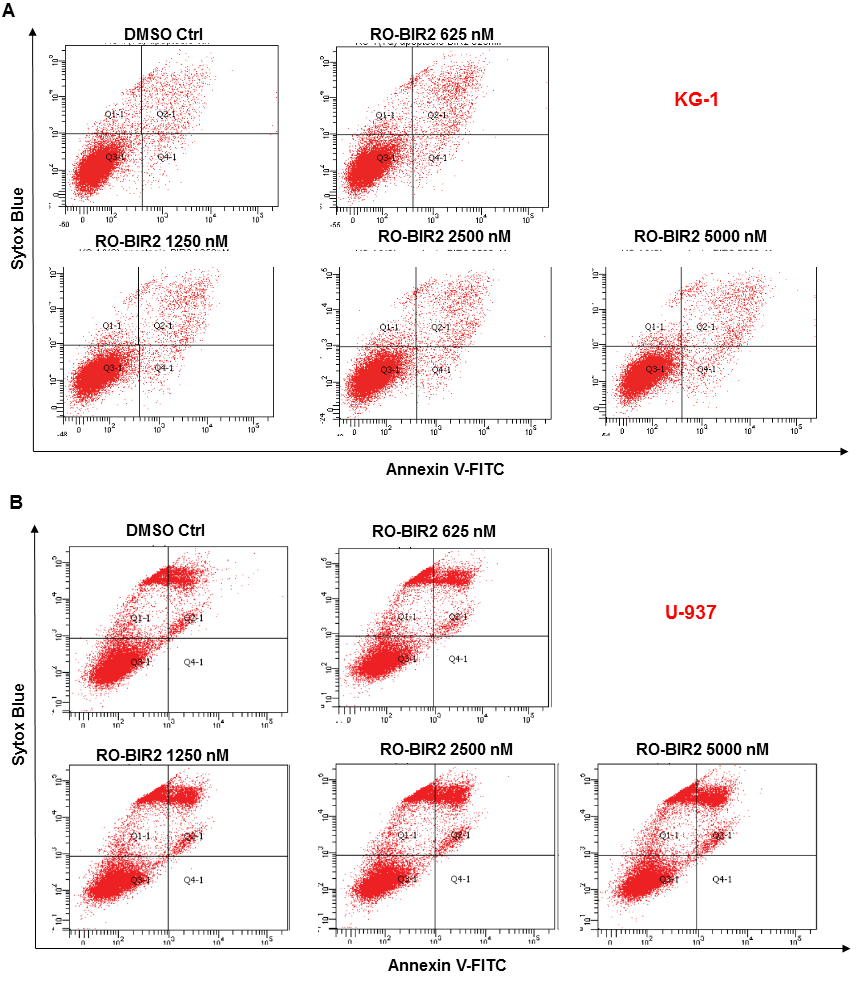


**Supplementary Figure S1: FACS analysis of apoptosis on KG-1 (A) and U-937 (B) cells treated with RO-BIR2.** Apoptosis assay of FACS on DMSO control, 625nM, 1250nM, 2500nM, 5000nM RO-BIR2 treated KG-1 and U-937 cells for 48 hours. Q2-1 and Q4-1 population represents cells undergoing apoptosis, and Q3-1 population represents viable cells.

**Supplementary Figure S2**

**
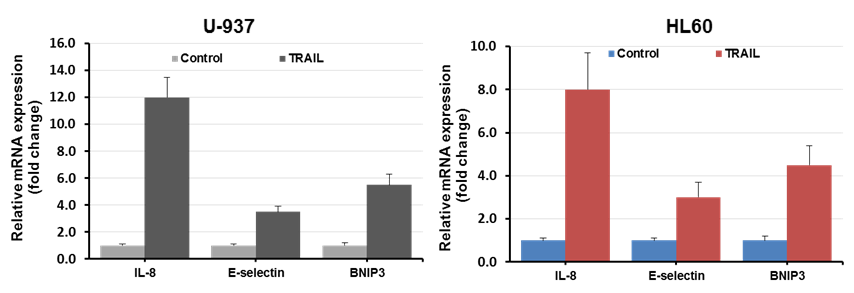
**

**Supplementary Figure S2. Quantification of TRAIL-induced genes in U-937 and HL60 cells by qRT-PCR.** U-937 and HL60 cells were treated with TRAIL (6.25 ng/ml) for 24 hours, then harvested for RNA extraction, followed by cDNA synthesis and qRT-PCR analysis. The relative expression of each gene in TRAIL-treated sample was normalized to its DSMO-treated sample (mean ± SD). The experiments were repeated thrice.

**Supplementary Figure S3**

**
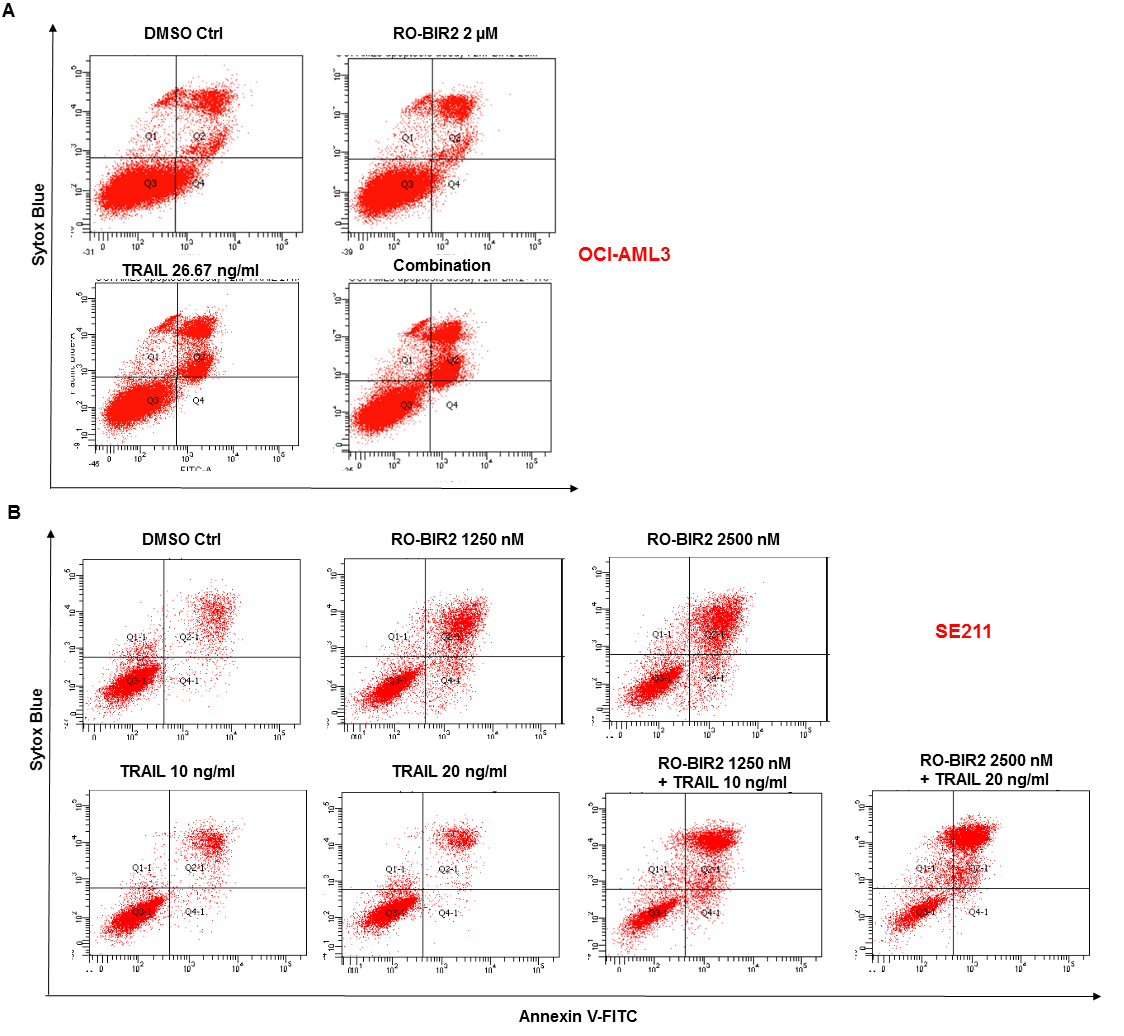
**

**Supplementary Figure S3.** **FACS analysis of specific apoptotic cell population of AML cell line OCI-AML3 (A) and primary AML cells from patient SE211 (B).**These cell were treated with either RO-BIR2 or TRAIL single agent or combination therapy of RO-BIR2 and TRAIL for 48 hours. Q2-1 and Q4-1 population represents cells undergoing apoptosis, and Q3-1 population represents viable cells.
